# Supplementary material for: COZOID: contact zone identifier for visual analysis of protein-protein interactions
Source: BMC Bioinformatics. 2018 Apr 6;19:125. doi: 10.1186/s12859-018-2113-6 (PMC5889581; doi:10.1186/s12859-018-2113-6)
Supplement: Supplementary file 2 — Software build. Executable binary file of the software tool. (ZIP 118,784 kb) [file 12859_2018_2113_MOESM2_ESM.zip › cozoid/platform/docs/testng-6.5.1-javadoc.zip!/org/testng/TestNGAntTask.Mode.html]

TestNGAntTask.Mode (TestNG)


---


|  |  |  |
| --- | --- | --- |
| **Overview** \| **Package** \| **Class** \| **Tree** \| **Deprecated** \| **Index** \| **Help** | |  |
| **PREV CLASS**   **NEXT CLASS** | **FRAMES**    **NO FRAMES**     **All Classes** |  |
| SUMMARY: NESTED \| ENUM CONSTANTS \| FIELD \| METHOD | DETAIL: ENUM CONSTANTS \| FIELD \| METHOD |  |


---


## org.testng Enum TestNGAntTask.Mode

```
java.lang.Object
  java.lang.Enum<TestNGAntTask.Mode>
      org.testng.TestNGAntTask.Mode
```

**All Implemented Interfaces:**: java.io.Serializable, java.lang.Comparable<TestNGAntTask.Mode>

**Enclosing class:**: TestNGAntTask

---

``` public static enum TestNGAntTask.Mode extends java.lang.Enum<TestNGAntTask.Mode> ```

---

**Enum Constant Summary**

`junit`

`mixed`

`testng`


| **Method Summary** | |
| --- | --- |
| `static TestNGAntTask.Mode` | `valueOf(java.lang.String name)`             Returns the enum constant of this type with the specified name. |
| `static TestNGAntTask.Mode[]` | `values()`             Returns an array containing the constants of this enum type, in the order they are declared. |

**Methods inherited from class java.lang.Enum**

`clone, compareTo, equals, finalize, getDeclaringClass, hashCode, name, ordinal, toString, valueOf`

**Methods inherited from class java.lang.Object**

`getClass, notify, notifyAll, wait, wait, wait`

**Enum Constant Detail**

### testng

```
public static final TestNGAntTask.Mode testng
```

---


### junit

```
public static final TestNGAntTask.Mode junit
```

---


### mixed

```
public static final TestNGAntTask.Mode mixed
```


**Method Detail**

### values

```
public static TestNGAntTask.Mode[] values()
```

:   Returns an array containing the constants of this enum type, in
    the order they are declared. This method may be used to iterate
    over the constants as follows:

    ```
    for (TestNGAntTask.Mode c : TestNGAntTask.Mode.values())
        System.out.println(c);
    ```

    :   **Returns:**: an array containing the constants of this enum type, in the order they are declared

---


### valueOf

```
public static TestNGAntTask.Mode valueOf(java.lang.String name)
```

:   Returns the enum constant of this type with the specified name.
    The string must match *exactly* an identifier used to declare an
    enum constant in this type. (Extraneous whitespace characters are
    not permitted.)

    :   **Parameters:**: `name` - the name of the enum constant to be returned. **Returns:**: the enum constant with the specified name **Throws:**: `java.lang.IllegalArgumentException` - if this enum type has no constant with the specified name: `java.lang.NullPointerException` - if the argument is null


---


|  |  |  |
| --- | --- | --- |
| **Overview** \| **Package** \| **Class** \| **Tree** \| **Deprecated** \| **Index** \| **Help** | |  |
| **PREV CLASS**   **NEXT CLASS** | **FRAMES**    **NO FRAMES**     **All Classes** |  |
| SUMMARY: NESTED \| ENUM CONSTANTS \| FIELD \| METHOD | DETAIL: ENUM CONSTANTS \| FIELD \| METHOD |  |


---
